# Codon-Optimized *Rhodotorula glutinis* PAL Expressed in *Escherichia coli* With Enhanced Activities

**DOI:** 10.3389/fbioe.2020.610506

**Published:** 2021-02-03

**Authors:** Feiyan Xue, Zihui Liu, Yue Yu, Yangjie Wu, Yuxin Jin, Mingfeng Yang, Lanqing Ma

**Affiliations:** Key Laboratory for Northern Urban Agriculture of Ministry of Agriculture and Rural Affairs, College of Bioscience and Resources Environment, Beijing University of Agriculture, Beijing, China

**Keywords:** phenylalanine ammonia lyase, codon optimization, *Rhodotorula glutinis*, *Escherichia coli*, tyrosine ammonia lyase

## Abstract

PAL (phenylalanine ammonia lyase) is important for secondary metabolite production in plants and microorganisms. There is broad interest in engineering PAL for its biocatalytic applications in industry, agriculture, and medicine. The production of quantities of high-activity enzymes has been explored by gene cloning and heterogeneous expression of the corresponding protein. Here, we cloned the cDNA of *Rhodotorula glutinis* PAL (*Rg*PAL) and introduced codon optimization to improve protein expression in *Escherichia coli* and enzyme activities *in vitro*. The *Rg*PAL gene was cloned by reverse transcription and named *pal*-wt. It had a full-length of 2,121 bp and encoded a 706-amino-acid protein. The *pal*-wt was inefficiently expressed in *E. coli*, even when the expression host and physical conditions were optimized. Therefore, codon optimization was used to obtain the corresponding gene sequence, named *pal*-opt, in order to encode the same amino acid for the *Rg*PAL protein. The recombinant protein encoded by *pal*-opt, named PAL-opt, was successfully expressed in *E. coli* and then purified to detect its enzymatic activity *in vitro*. Consequently, 55.33 ± 0.88 mg/L of PAL-opt protein with a specific activity of 1,219 ± 147 U/mg and *K*_m_ value of 609 μM for substrate L-phenylalanine was easily obtained. The enzyme protein also displayed tyrosine ammonia lyase (TAL)–specific activity of 80 ± 2 U/mg and *K*_m_ value of 13.3 μM for substrate L-tyrosine. The bifunctional enzyme *Rg*PAL/TAL (PAL-opt) and its easy expression advantage will provide an important basis for further applications.

## Introduction

**Figure 1:**
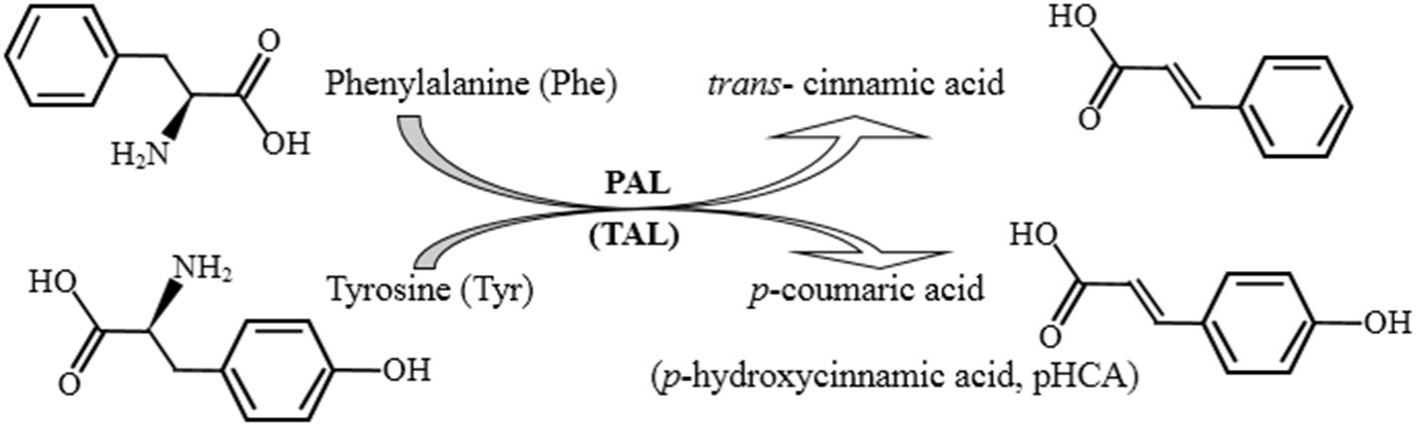
The enzyme reaction mechanism (deamination) of PAL (TAL).

Phenylalanine ammonia lyase (PAL, EC 4.3.1.5) is the first enzyme of the general phenylpropanoid pathway catalyzing ammonia elimination from phenylalanine (Phe) to give *trans*-cinnamic acid, or tyrosine (Tyr) deamination to form *p*-coumaric acid (*p*-hydroxycinnamic acids), indicating its additional tyrosine ammonia lyase (TAL) activity (Figure 1) (Jun et al., 2018). It plays an important role in the synthesis of secondary metabolites with high biological value and has been of great interest in the food industry, agriculture, and medicine (Wang et al., 2016; Jun et al., 2018; Levy et al., 2018; Lin et al., 2018; Otto et al., 2019; Mays et al., 2020). Initially discovered as a plant enzyme, PAL has also been subsequently found in some microorganisms (Ogata et al., 1967; Barron et al., 2017; Levy et al., 2018). Among the PAL-producing microorganisms, yeasts, especially red yeasts, have garnered great interest regarding potential enzyme production (MacDonald and D'Cunha, 2007; Cui et al., 2014). Furthermore, the genus *Rhodotorula* has been the primary commercial source of enzyme (D'Cunha et al., 1996; D'Cunha, 2005; Cui et al., 2008; Barron et al., 2017). Studies on improving *Rhodotorula* PAL stability and activity have been focused. To increase enzymatic stability, the immobilized *Rhodotorula* PAL has been evaluated (Cui et al., 2015, 2017). To increase enzymatic activity, the cloning and heterogeneous expression of *Rhodotorula* PAL in recombinant *Escherichia coli* have typically been used (Cui et al., 2008; Jia et al., 2008; Babich et al., 2013; Zhu et al., 2013; Vargas-Tah et al., 2015; Rowles et al., 2016; Levy et al., 2018). Some methods, such as induction by the addition of amino acids, organic solvents, and surfactants (Cui et al., 2008), directed evolution by site-specific mutagenesis (Rowles et al., 2016; Mays et al., 2020), and coexpression of 3-deoxy-d-*arabino*-heptulosonate-7-phosphate synthase and transketolase (Vargas-Tah et al., 2015), have been further used to enhance recombinant PAL production. Here, we cloned the full-length *R. glutinis* PAL (*Rg*PAL) gene and provided an efficient expression of the recombinant enzyme in *E. coli* by codon optimization. This low-cost and easy method reported here for obtaining abundant recombinant *Rg*PAL with high activity offers an effective and sustainable PAL production source.

## Materials and Methods

### Microbial Strains, Plasmids, and Reagents

The strains used in this work included *Rhodotorula glutinis* CGMCC2258 and *E. coli* BL21 (DE3), Rosetta-Gami 2 (DE3), and DH5α. The plasmids PMD-18T and pET-30a were used for gene cloning and expression, respectively. TransStart FastPfu DNA polymerase DNA kit, Plasmid Extraction kit, HisPur™ Ni-NTA Resin, and BCA Protein Assay kit were purchased from Beijing Solarbio Science & Technology Co., Ltd (Beijing, China). The restriction endonucleases (*Hin*dIII and *Eco*RI) and the rapid amplification of cDNA end (RACE) kits (3′-Full RACE Core Set and 5′-Full RACE) were purchased from Takara (Shiga, Japan). Yeast extract and peptone were purchased from Oxoid (Basingstoke, Hampshire, UK). All solvents for high-performance liquid chromatography (HPLC) analysis were purchased from Fisher Scientific (Fair Lawn, NJ, USA). All chemicals, including standards of *trans*-cinnamic acid and *p*-coumaric acid, were purchased from Sigma–Aldrich (St. Louis, MO, USA) unless indicated otherwise.

### Gene Cloning and Plasmid Construction

Total RNA of *R. glutinis* was extracted with TRIzol (Zhu et al., 2013). The first-strand cDNA was synthesized using a RevertAid First Strand cDNA Synthesis Kit (Thermo Scientific, Waltham, MA, USA). Based on the nucleotide sequences of *Rhodotorula* PAL published in NCBI (GenBank accession no. KF770992.1, DQ013364.1, and X13094.1), the specific primers F1 and R1 were designed to match the conserved region. F2 (gene-specific primer based on the amplified sequence above) and R2 (3′ RACE out primer based on the 3′ RACE adaptor) were applied for 3′ RACE to obtain the 3′ fragment. Degenerate oligonucleotide-primed polymerase chain reaction (DOP-PCR) further proceeded using the F3 and R3 primers. The primers F4, R4, F5, and R5 were used in a nested PCR strategy for the 5′ RACE to obtain the 5′ fragment. Based on the sequence information obtained above, the specific primers F6 and R6 were further designed to amplify the full-length cDNA, which was named *pal*-wt. All of the amplified fragments were subcloned into the plasmid PMD-18T for identification by sequencing. The cloning process is shown in Supplementary Figure 1.

To construct the recombinant plasmid, the F7 and R7 primers containing the restriction endonuclease sites of *Hin*dIII and *Eco*RI, respectively, were used to amplify *pal*-wt. All primers used are shown in Table 1. The amplified products were purified and ligated into the PMD-18T vector to get PMD-18T-*pal*-wt for identification by restriction enzyme digestion and sequencing. The expected *pal*-wt fragment was extracted and ligated into the pET-30a vector with the His6-tag that had been previously digested with *Hin*dIII and *Eco*RI to yield the recombinant plasmid pET-30a-*pal*-wt. The empty vector and ligated products were then transformed into *E. coli* BL21 (DE3) and *E. coli* Rosetta-Gami 2 (DE3) for expression identification.

**Table 1:** Primers used in this study.

**Primer**	**Sequence 5^**′**^ → 3^**′**^**	**Note**	
F1	GTCAAGGTCAAGGACGACGAGG		
		Highly conserved region amplification	
R1	CGTTGAGCATCTCGGTGAGCT		
F2	ATCGACCTCGAGAACAAGATGACC	Gene-specific primer (GSP1)	3′ RACE
R2	TACCGTCGTTCCACTAGTGATTT	3′ RACE out primer	
F3	GCAGTNTAYGGNCNACNGGNTTYGG	degenerate primers	DOP-PCR
R3	TGCATACGGAACTATCATGGTCTAC	Gene-specific primer (GSP2)	
F4	CATGGCTACATGCTGACAGCCTA	5′ RACE Out Primer	5′ RACE
R4	AGTGACCACGCGTCAACGAGTTG	Gene-specific primer (GSP3)	
F5	CGCGGATCCAACAGCCTACTGATGATCAGTCGATG	5′ RACE Inner Primer	
R5	GTCTGCCGAG CCACCGAAGC	Gene-specific primer (GSP4)	
F6	ATGGCCCCCTCTGTGGACTCGAT		
		Full-length of *pal*-wt amplification	
R6	TTAGGCCATCATCTTGACGAGGACG		
F7	TGGAATTCCATGGCCCCCTCT		
		Recombinant plasmid construction	
R7	GCAAGCTTGTTAGGCCATCATCTTG		

*N = A/C/G/T, Y = C/T. The underlined nucleotides denote restriction endonuclease sites*.

### Codon Optimization

Without changing the corresponding amino acid sequence, the *pal*-wt gene was optimized by replacing the codons to balance its G + C content and avoid restriction endonuclease sites, the ribosome binding site, and the rare codons of *E. coli*. Named *pal*-opt, the optimized gene introduced with the restriction sites (*Hin*dIII and *Eco*RI) was synthesized by Synbio Technologies (Suzhou, China). The synthetic *pal*-opt gene was digested and ligated into the pET-30a vector to generate the recombinant plasmid pET-30a-*pal*-opt. The empty vector and resulting plasmid were transformed into *E. coli* BL21 (DE3) for expression identification.

### Protein Expression and Purification

The recombinant *E. coli* with pET-30a, pET-30a-*pal*-wt, or pET-30a-*pal*-opt was cultured in lysogeny broth (LB) medium at 37°C until the expected OD_600_ was reached. Expression of the recombinant gene was induced by the addition of 0.5 mM Isopropyl β-D-Thiogalactoside (IPTG), followed by an incubation process at the designed temperature and time. Sodium dodecyl sulfate–polyacrylamide gel electrophoresis (SDS-PAGE) using 12% SDS–polyacrylamide gels was performed to determine the approximate molecular mass and expression quantity of the recombinant PAL. The optimal induction conditions achieved above were further used for protein expression in large quantities. Centrifugation (10,000 × *g*, 10 min, 4°C) proceeded after the cells were disrupted by sonication (40 × 5 s) in ice bath. The supernatant was passed through *ProteiIso* Ni-NTA resin. The target PAL-opt tagged with 6 × His was purified by eluting with different imidazole concentrations (20, 200, and 500 mM). The collected fractions were analyzed by SDS-PAGE. Protein samples were then concentrated in ultracentrifuge tubes (MM Amicon Uitracel-30K) and purified with a PD-10 desalting column (Amersham Bioscience, Little Chalfont, UK). The purified PAL-opt was stored at 4°C until further analysis and application.

### Enzyme Yield and Activity Assay

The enzyme yield was assessed by calculating the purified protein content per liter of culture broth. The protein concentration was measured with a BCA Protein Assay kit after purification.

PAL activity was assayed by monitoring the formation of *trans*-cinnamic acid catalyzed by the purified protein. The reaction mixture included 4 μg purified protein and 2.5 mL × 50 mM l-Phe, with 25 mM Tris-HCl buffer (pH 8.8) to obtain a total volume of 5 mL. The reaction was performed at 40°C for 30 min, with 5 mL methanol added to be terminated. The reaction product was evaporated at 60°C, following which it was redissolved in 5 mL methanol. After centrifugation (12,000 × *g*, 10 min), the supernatant was filtered through a nylon membrane (0.22 μm) to obtain the sample for HPLC analysis. Five microliters of sample was analyzed by HPLC at 290 nm with a C_18_ column (4.6 × 250 mm, 5 μm, Agela Innoval, CA, USA) and a gradient elution using a mobile phase comprising (A) methanol and (B) 1.5% acetic acid in water, starting at 30% A and increasing linearly to 50% until 20 min. One unit of enzyme catalyzed 1.0 μmol of *trans*-cinnamic acid synthesis per minute under the assay conditions. The assay of Michaelis–Menten kinetic parameter (*K*_m_) was performed with different concentrations of l-Phe from 1 to 40 mM. To obtain the optimal reaction pH and temperature, the PAL activity was measured at different temperatures (from 30 to 60°C) and pH values (from 6 to 10).

TAL activity was assayed by monitoring the formation of *p*-coumaric acid catalyzed by the purified protein. The reaction mixture included 10 μg purified protein and 1 mL × 50 mM Tyr, with 25 mM Tris-HCl buffer (pH 7.5) to obtain a total volume of 5 mL. The reaction was performed at 40°C for 30 min, with 5 mL methanol added to terminate the reaction. The reaction product was freeze-dried and extracted with 5 mL methanol. The extract solution was centrifuged (10,000 × *g*, 3 min), and the supernatant was filtered through a nylon membrane (0.22 μm) to prepare the sample for HPLC analysis. Ten microliters of sample was analyzed by HPLC at 310 nm with an isocratic elution using a mobile phase comprising 20% A (acetonitrile) and 80% B (3.5% acetic acid in water) over 10 min. One unit of enzyme catalyzed 1.0 μmol of *p*-coumaric acid synthesis per minute under the assay conditions. The assay of *K*_m_ was performed with different concentrations of l-Tyr from 1 to 25 mM. To obtain the optimal reaction pH and temperature, the TAL activity was measured at different temperatures (from 30 to 60°C) and pH values (from 6 to 10).

## Results and Discussion

### Results of Gene Cloning and Recombinant Strain Construction

Some *Rhodotorula* PAL gene sequences have been reported in the NCBI database. However, it is still not easy to obtain the full-length cDNA because of the significant differences in sequences, especially at the two ends 3′ and 5′ (Supplementary Figure 2).

**Figure 2:**
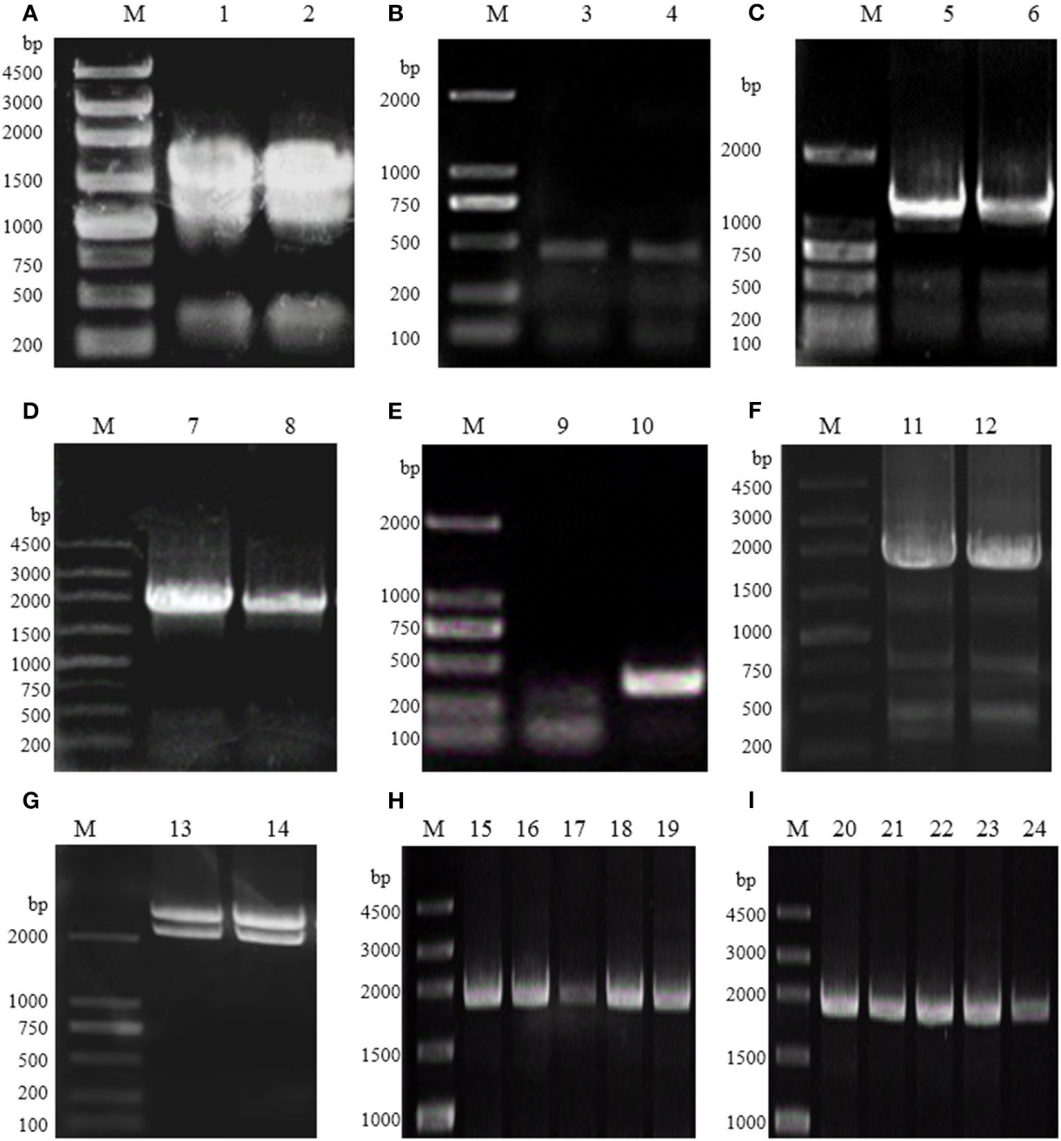
Electrophoresis analysis of *R. glutinis* PAL gene cloning and recombinant strain construction. **(A)** Total RNA from *R. glutinis*. **(B)** The conserved region of PAL. **(C)** Product of 3′ RACE. **(D)** Product of DOP-PCR. **(E)** Product of 5′ RACE. **(F)** Full-length of *pal*-wt. **(G)** Double-enzyme digestion of PMD-18T-*pal*-wt. **(H)** Products of colony PCR of recombinant *E. coli* BL21 (DE3). **(I)** Products of colony PCR of recombinant strains *E. coli* Rosetta-Gami 2 (DE3). M, DNA Marker; 1–2, total RNA; 3–12, PCR products; 13–14, double-enzyme digestion products; 15–24, colony PCR products.

Therefore, the full-length *Rg*PAL cDNA was obtained using reverse transcription–PCR, DOP-PCR, and RACE technology. The total RNA extracted from *R. glutinis* had three obvious bands (28S, 18S, and 5S), as shown in Figure 2A. The quality and quantity assessment of the extracted RNA indicated an OD_260_/OD_280_ of 1.9 and a concentration of 2.5 μg/μL. As shown in Figure 2B, a 450-bp fragment was obtained based on the conserved sequence of known PALs in *Rhodotorula* (GenBank accession no. KF770992.1, DQ013364.1, and X13094.1). Subsequently, fragments of 1,252, 1,922, and 357 bp were amplified using 3′ RACE, DOP-PCR, and 5′ RACE (Figures 2C–E). The full-length *Rg*PAL cDNA sequence, named *pal*-wt, was eventually isolated (Figure 2F). Its recombinant plasmid pMD-18T-*pal*-wt was identified, as shown in Figure 2G, after digestion by *Hin*dIII and *Eco*RI. The recombinant strains *E. coli* BL21 (DE3) and *E. coli* Rosetta-Gami 2 (DE3) with plasmid pET-30a-*pal*-wt were constructed and identified by colony PCR, as indicated in Figures 2H,I.

The results of sequencing analysis indicated that the full-length of *pal*-wt contains an open reading frame (ORF) of 2,121 bp (GenBank accession no. MG712805). It encodes 706 amino acid residues with a predicted protein molecular mass of 75.5 kDa and an isoelectric point of 6.8. It shares the highest DNA (88.75%) and amino acid (96.35%) identity with sequence no. KF770992.1, according to multiple sequence alignment performed by DNAMAN software. Its protein sequence also contains the reported *Rhodotorula* PAL signature motif of “GTISASGDLSPLSYIAA” (Hyun et al., 2011; Yun et al., 2015) positioned between amino acids 209 and 225 (Supplementary Figure 3). The conserved active site of cyclized tripeptide Ala^213^-Ser^214^-Gly^215^, constructing a prosthetic 4-methylidene imidazole-5-one (MIO) group, identified in the ORF sequence also demonstrated that *pal*-wt is a *Rg*PAL gene, the protein of that could be subsequently expressed (Lin et al., 2018).

### Results of Protein Expression and Codon Optimization

**Figure 3:**
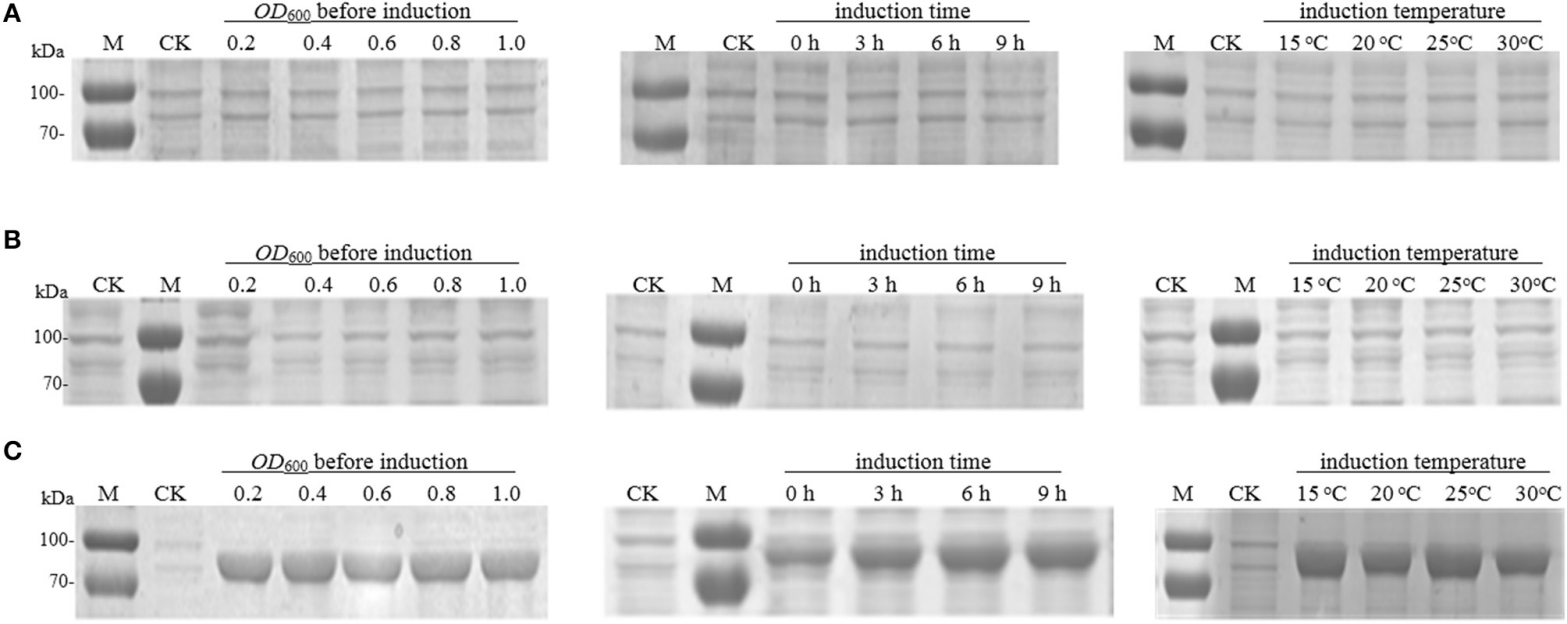
SDS-PAGE analysis of *R. glutinis* PAL expression. **(A)** *pal*-wt expression in *E. coli* BL21 (DE3). **(B)** *pal*-wt expression in *E. coli* Rosetta-Gami 2 (DE3). **(C)** *pal*-opt expression in *E. coli* BL21 (DE3). M, protein marker; CK, control (cell with empty vector induced at 30°C and OD_600_ = 0.6 for 6 h).

The recombinant strain *E. coli* BL21 (DE3) harboring the plasmid pET-30a (as control) or pET-30a-*pal*-wt was induced by the addition of IPTG to identify *Rg*PAL expression. The SDS-PAGE analysis results (Figure 3A) indicated that the target protein did not appear at all, even after optimizing the cell concentration before induction, the cultivation time after induction, and the cultivation temperature during induction. The *E. coli* BL21 (DE3) was temporarily suspended and considered to be unable to express *Rg*PAL due to codon bias problems. Rosetta™ host strains, as BL21 derivatives, are designed to enhance the expression of eukaryotic proteins that contain codons rarely used in *E. coli* (Yin et al., 2007; Rai et al., 2020). Subsequently, we changed the host to *E. coli* Rosetta-Gami 2 (DE3) with an abundance of tRNAs for rare codons and expressed the protein under different induction conditions with respect to *Rg*PAL. However, none of these experiments resulted in the target protein band (Figure 3B).

Being confronted with these results, the *pal*-wt sequence was further analyzed in detail based on our previous experience of recombinant protein expression (Xue et al., 2016), reported codon usage tables (Athey et al., 2017), and considerations in the use of codon optimization for recombinant protein expression (Mauro and Chappell, 2018). According to the sequence analysis, the G + C content and rare codons were considered as potential problems. The total G + C content in the *pal*-wt gene was as high as 65.1%, with some higher partial G + C contents. Many rarely used codons in *E. coli*, such as AGG, CGA, CGG, GGA, and CCC, existed in the *pal*-wt sequence (Table 2). Among them, 10 of the 36 arginine codons are rare in *E. coli*. Mistranslation errors associated with the rare arginine codon CGG in *E. coli* have already been observed (McNulty et al., 2003). Consequently, codon optimization was applied without changing the amino acid sequence. The G + C content was reduced to 49.6%, and the rare codons were avoided in the optimized sequence, which was named *pal*-opt (GenBank accession no. MK748987). The *pal*-opt gene was ligated into vector pET-30a to generate the plasmid pET-30a-*pal*-opt and then transformed into *E. coli* BL21 (DE3) competent cells. The resulting strain *E. coli* BL21 (DE3) harboring the plasmid pET-30a (as control) or pET-30a-*pal*-opt was induced by addition of IPTG to identify PAL-opt expression. The presence of a protein band of ~75.5 kDa in the IPTG-induced culture was in accordance with the expected size of the *Rg*PAL protein (Figure 3C). Considerable amounts of recombinant protein were easily produced with little influence of cell concentration (OD_600_: 0.2–1.0) before induction, at a cultivation temperature of 15–30°C during induction and after 3 h of cultivation time after induction. A certain amount of the PAL-opt protein was produced under control of a T7 promoter even in the absence of IPTG, which is consistent with reported observations (Hartinger et al., 2010).

**Table 2:** The codons of *pal*-wt rarely used in *E. coli*.

**Amino acid**	**Rare codon(s)**	**Occurrences**
Arginine	AGG	1
	CGA	1
	CGG	8
Glycine	GGA	4
Proline	CCC	5

### Results of Protein Purification and Enzyme Characterization

**Figure 4:**
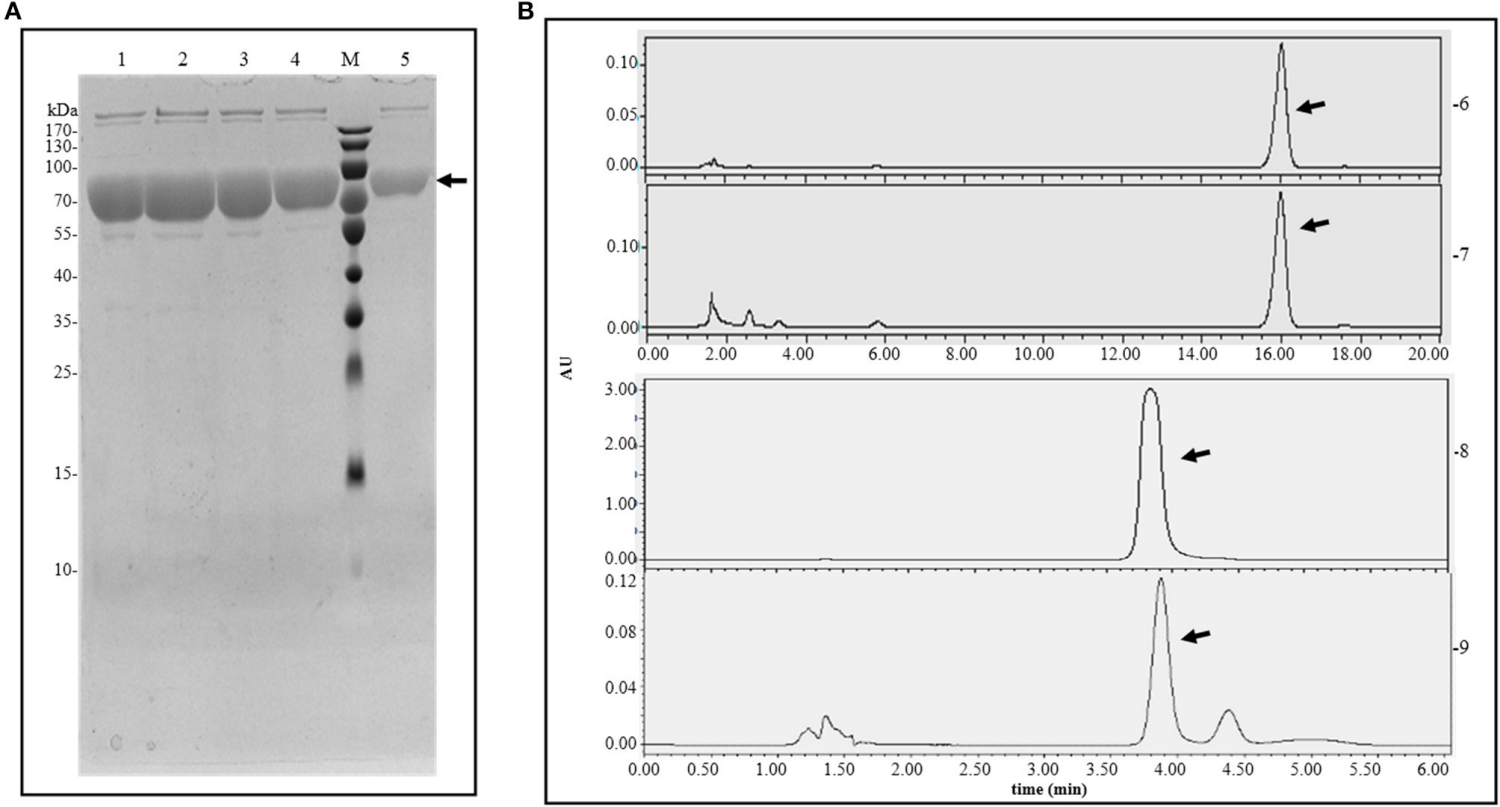
Protein purification and enzyme catalysis results. **(A)** Protein purification analyzed by SDS-PAGE. **(B)** Enzyme catalysis analyzed by HPLC. M, protein marker; 1–5, purified protein; 6, *trans*-cinnamic acid standard; 7, products of enzyme as PAL catalysis; 8, *p*-coumaric acid standard; 9, products of enzyme as TAL catalysis. The arrow points to the target band or peak.

After expressing the *E. coli* BL21 (DE3) protein harboring the plasmid pET-30a-*pal*-opt, purification was conducted using nickel column (*ProteiIso* Ni-NTA Resin) affinity chromatography. The SDS-PAGE analysis showed that the target protein eventually appeared in a single band of ~75.5 kDa (Figure 4A). The purified protein was measured by a BCA kit, resulting in a yield of 55.33 mg/L (Table 3). The enzyme activity of the purified protein was measured using l-Phe or l-Tyr as the substrate. The products of the enzyme catalysis reaction were detected by HPLC using *trans*-cinnamic acid or *p*-coumaric acid as the standards (Figure 4B). Therefore, PAL-opt is actually *Rg*PAL/TAL as it displayed both PAL and TAL activities, leading to the formation of *trans*-cinnamic acid and *p*-coumaric acid.

**Table 3:** Enzyme activity comparison of recombinant *Rhodotorula* PAL or TAL.

**Protein yield (mg/L)**	**Activity**	**Reaction system**		**References**
		**Tris-HCl buffer**	**Temperature**	
N/A	PAL: 4.2 U/mg	25 mM, pH 8.0	40°C	Zhu et al., 2013
77.4	TAL:7.53 U/mg	50 mM, pH 8.5	Room temperature	Vannellia et al., 2007
20–25	PAL: 307.28 nmol/min/mg	100 mM, pH 8.8	N/A	Rowles et al., 2016
55.33	PAL: 1,219 U/mg	25 mM, pH 8.8	40°C	This study
	TAL: 80 U/mg	25 mM, pH 7.5		

*N/A, not reported*.

**Figure 5:**
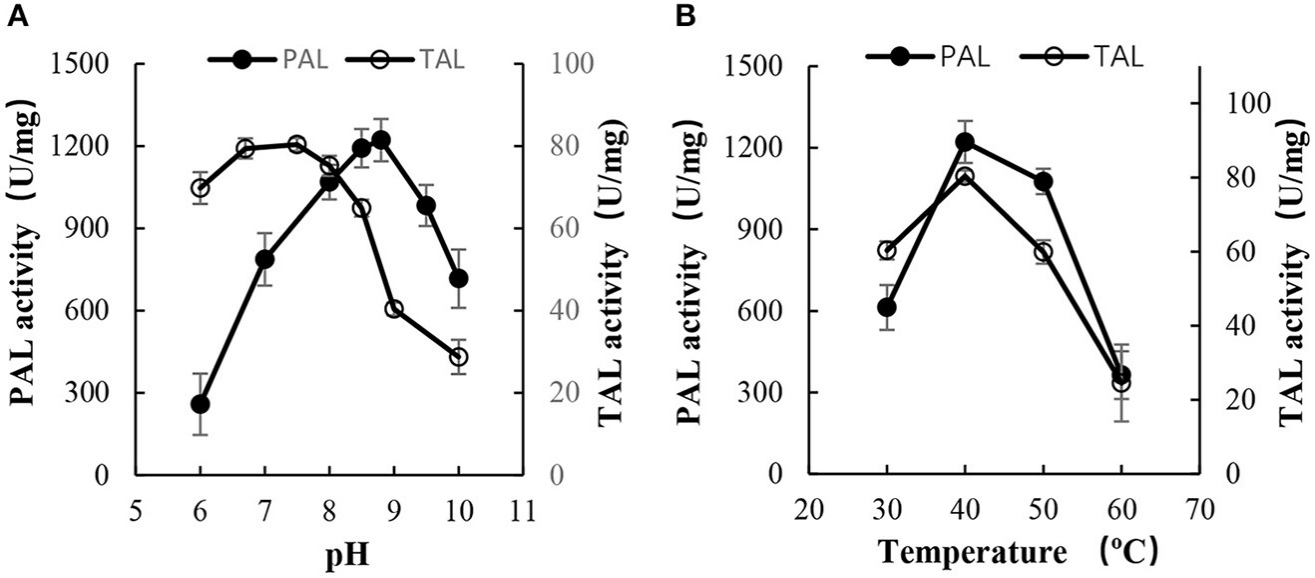
Effect of pH and temperature on the activity of Rg PAL/TAL. **(A)** Effect of pH (pH 6–7, 25 mM sodium acetate buffer; pH 7–9, 25 mM Tris-HCl buffer; pH 9–10, 25 mM sodium carbonate buffer). The reactions were performed at 40°C for 30 min to determine enzyme activity. **(B)** Effect of temperature. The reactions were performed for 30 min at pH 8.8 to determine PAL activity while at pH 7.5 to determine TAL activity. Each value is the mean ± SE for *n* = 3.

As shown in Figure 5, PAL-opt exhibited a considerable high activity and relative stability. To display the activity of PAL and TAL, the optimum pH values were 8.5–9.0 and 6.5–7.5, respectively (Figure 5A), and the optimum temperature was 40°C (Figure 5B). The activities of PAL and TAL were as high as 1,219 ± 147 and 80 ± 2 U/mg, respectively.

As summarized in Table 3, the specific enzyme activities of PAL and TAL of the purified PAL-opt protein were as high as 1,219 ± 147 U/mg (the equivalent of 67,045 U/L of culture broth) and 80 ± 2 U/mg, respectively. However, cell-free extracts of *R. glutinis* only possessed 0.0241 and 0.0143 U/mg of PAL and TAL activities (Vannellia et al., 2007). Different technologies application resulted in the improvement of the enzyme activity. Zhu et al. obtained the highest *Rg*PAL activity of 4.2 U/mg of purified protein in recombinant *E. coli* BL21 (DE3) at an optimal pH and temperature (Zhu et al., 2013). Rowles et al. engineered *Rhodotorula graminis* PAL in recombinant *E. coli* BL21 (DE3) to obtain an activity of 307.28 nmol/min/mg (the equivalent of 6.15–7.68 U/L of culture broth) by site-directed mutagenesis (Rowles et al., 2016). The specific activity of *Rg*TAL was improved by up to 7.53 U/mg via heterologous expression in *E. coli* W3110 (Vannellia et al., 2007). Here, the improvement of enzyme activities was contributed by purified protein in recombinant *E. coli* BL21 (DE3) using codon optimization and the optimum reaction system.

A wide range of specific activity and *K*_m_ values for substrates l-Phe and l-Tyr has been observed in the studies of several enzymes displaying both PAL and TAL activities (Vargas-Tah and Gosset, 2015). A comparison of *Rhodotorula* PAL/TAL (Table 4) also revealed the difference in *K*_m_ values for substrates l-Phe and l-Tyr. The difference was more significant for the enzyme of recombinant protein than the original enzyme from cell-free extract. Even if all belong to the recombinant protein of *Rg*PAL/TAL, the *K*_m_ values for substrates l-Phe and l-Tyr were quite different. Obviously, the *K*_m_ for TAL observed in this study indicated its high affinity to l-Tyr substrate, which would be valuable to produce a wide range of aromatic compounds that using l-Tyr or its derivative as a precursor (Shen et al., 2020).

**Table 4:** Kinetic parameter comparison of *Rhodotorula* PAL/TAL.

**Gene Source**	***K***_****m****_ **(μM)**		**Note**	**References**
	**PAL (l-Phe)**	**TAL (l-Tyr)**		
*Rhodotorula rubra*	446	220	Cell-free extract	Vannelli et al., 2007
*Rhodotorula graminis*	448	154		Vannelli et al., 2007
*Rhodotorula minuta*	584	212		Vannelli et al., 2007
*Rhodotorula glutinis*	518	209		Vannelli et al., 2007
	400	110	Recombinant protein	Vannellia et al., 2007
	1,340	560		Zhu et al., 2013
	1,720	550		Liang et al., 2016
	609	13.3	Recombinant protein Codon optimization	This study

**Figure 6:**
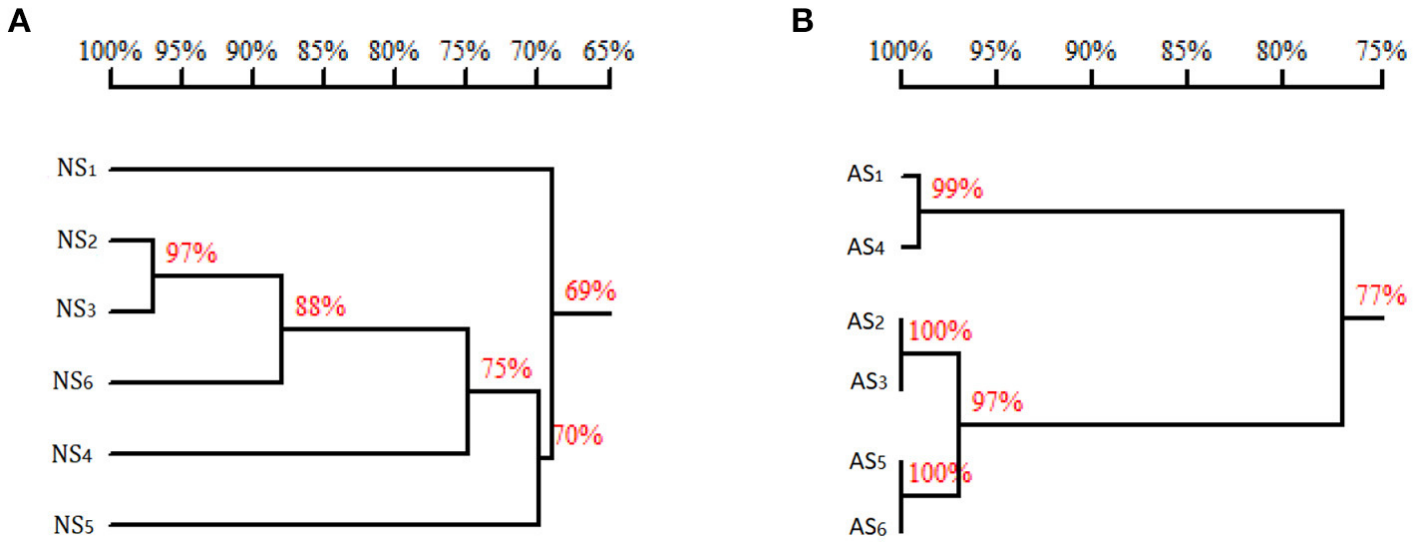
Homology tree of *Rhodotorula* PAL (TAL). **(A)** The homology of the nucleotide sequences. **(B)** The homology of the amino acid sequences. NS, nucleotide sequence; AS, amino acid sequence; 1, sequence of GenBank KF765779.1 (*Rhodotorula glutinis*); 2, sequence of GenBank KF770992.1 (*R. glutinis*); 3, sequence of GenBank X13094.1 (*Rhodotorula mucilaginosa*); 4, sequence of GenBank X12702.1 (*Rhodotorula toruloides*); 5, sequence of GenBank MK748987.1 (*R. glutinis, pal*-opt in this study); 6, sequence of GenBank MG712805.1 (*R. glutinis, pal*-wt in this study). Homology tree was constructed by DNAMAN.

Why are there such significant differences in enzyme activities (Table 3) and *K*_m_ values (Table 4) of PALs originated from the same genus of red yeast and expressed in *E. coli*? A high production and purity of the recombinant protein contributed by codon optimization (Figure 3) to remove the obstacle of nucleotide sequence (Figure 6A) as observed in this study should be one reason. An optimum reaction system mentioned above (Figure 5 and Table 3) should be another. Moreover, the differences in amino acid sequences will lead to the different enzyme activity and specificity (Figure 6B). Just as Zhu et al. (2013) reported that although *R. glutinis* is an anamorph of *Rhodosporidium toruloides*, the amino acid sequences of PALs are not the same (about 74% identity).

## Conclusion

Given increased focus on health, industry demands, agriculture development, and biotechnology, high-activity PAL is becoming increasingly desirable. Recombinant PAL is believed to be a potential source because of its optional gene origin and protein expression host, rational design, and directed evolution. PAL genes originating in the genus *Rhodotorula* have been shown to possess significant enzyme activity. Some PAL also displays TAL activity, in the term of PAL/TAL. In this study, when a full length of gene was cloned, an effective *Rg*PAL expression method that does not require special physiological conditions was herein developed by codon optimization, providing *Rg*PAL/TAL with high activity and high affinity to l-Tyr for further applications.

## Data Availability Statement

The datasets presented in this study can be found in online repositories. The names of the repository/repositories and accession number(s) can be found in the article/Supplementary Material.

## Author Contributions

FX: conceptualization, methodology, investigation, resources, validation, supervision, funding acquisition, and writing–original draft preparation. ZL: codon optimization of *Rg*PAL and protein expression. YY: gene cloning of *Rg*PAL and recombinant plasmids construction. YW: enzyme assessment of *Rg*PAL. YJ: kinetic characterization of *Rg*PAL. MY: software, validation, and writing–reviewing and editing. LM: supervision, resources, and writing–reviewing and editing. All authors contributed to the article and approved the submitted version.

## Conflict of Interest

The authors declare that the research was conducted in the absence of any commercial or financial relationships that could be construed as a potential conflict of interest.
