# Supplementary material for: Codon-Optimized Rhodotorula glutinis PAL Expressed in Escherichia coli With Enhanced Activities
Source: Front Bioeng Biotechnol. 2021 Feb 3;8:610506. doi: 10.3389/fbioe.2020.610506 (PMC7886678; doi:10.3389/fbioe.2020.610506)
Supplement: Supplementary file 1 [file Data_Sheet_1.PDF]

Supplementary material

Codon-optimized *Rhodotorula glutinis* PAL expressed in *Escherichia coli* with enhanced activities

Feiyan Xue*, Zihui Liu, Yue Yu, Yangjie Wu, Yuxin Jin, Mingfeng Yang, Lanqing Ma*

Key Laboratory for Northern Urban Agriculture of Ministry of Agriculture and Rural Affairs,

College of Bioscience and Resources Environment, Beijing University of Agriculture, Beijing 102206, China

***corresponding authors:** feiyanxue@bua.edu.cn; lqma@bua.edu.cn

Supplementary figures

Figure legends

Fig. S1. Schematic overview of *Rg*PAL gene cloning.

Black bold line represented the gene of RgPAL to be cloned.

Fig. S2. Multiple sequence aligment of *Rhodotorula* PAL gene.

Nucleotide indentity is shown with black shading, and nucleotide similarity is shown with green shading. Sequences aliged were *Rhodotorula glutinis* DQ013364.1, *Rhodotorula glutinis* KF77091.1, and *Rhodotorula mucilaginosa* X13094.1.

Fig. S3. Multiple sequence aligment of PAL encoded by *Rhodotorula glutinis* KF77091.1 and *Rhodotorula glutinis* MG712805.1 .

The *Rhodotorula* PAL signature motif is indicated in red box. The cyclized active site tripeptide Ala^{213}-Ser^{214}-Gly^{215} (MIO) is underlined. The histidine (position 136) is indicated with solid triangles.

Fig. S1

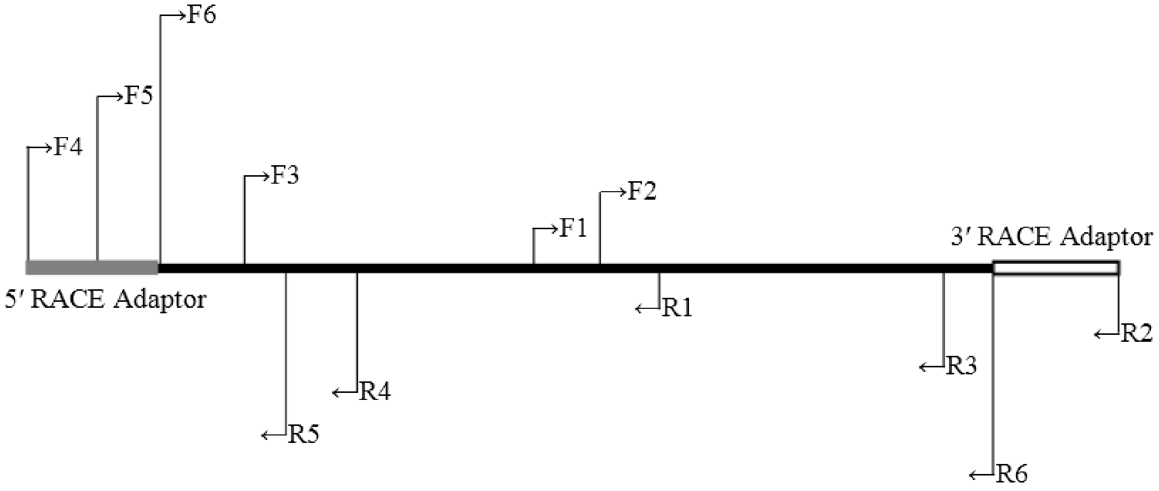

Figure S2

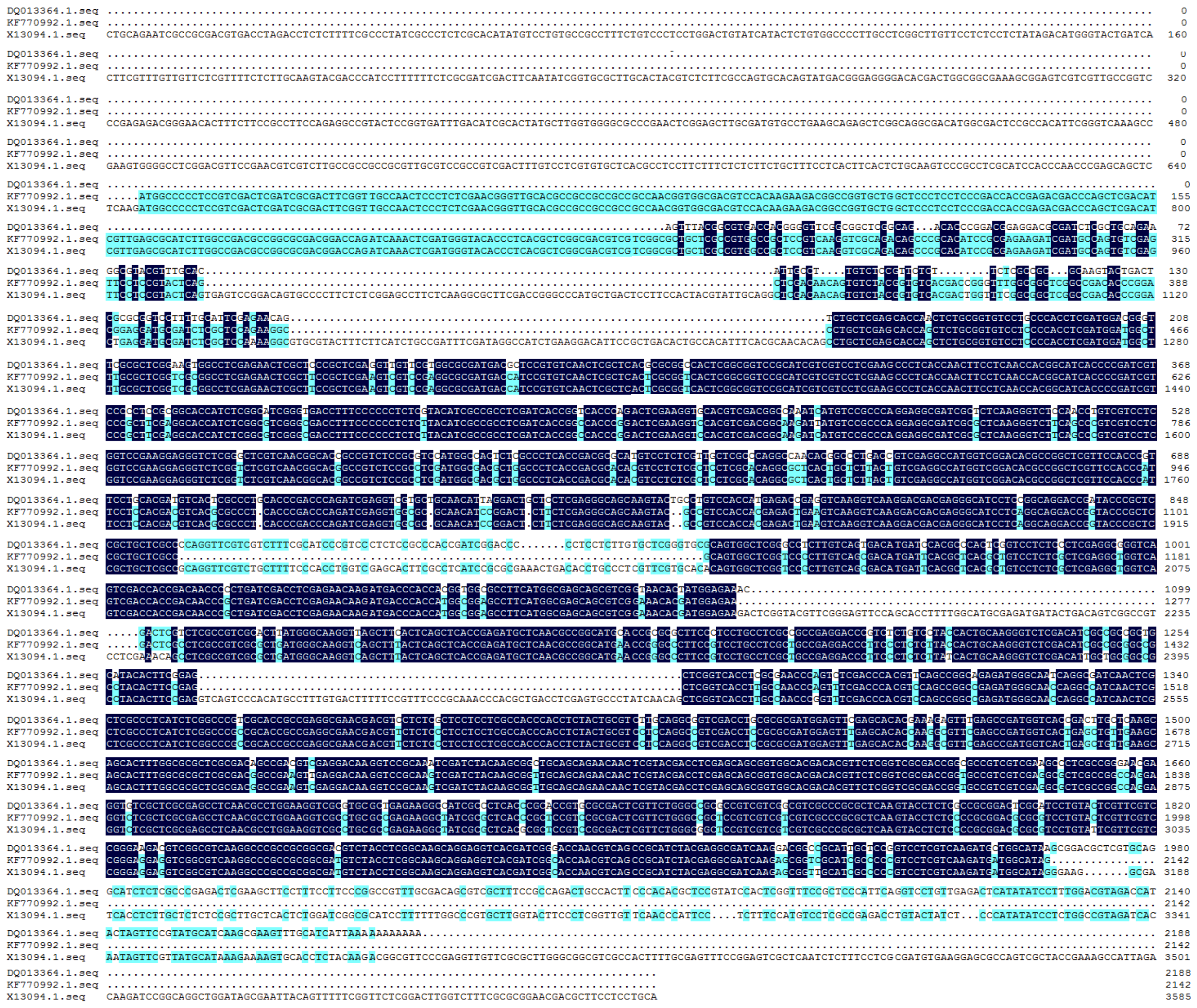

Fig. S3

```
AA-KF770992.1.seq   MAPSVDSIATSVANSLSNGLHAAAAANGGDVHKKTAGAGSLLPTTETTQLDIVERILADAGATDQIKLDGYTLTLGDVVGAARRGRSVKVADSPHIREKIDASVEFLRTQLDNSVYGVTT   120
AA-MG712805.1.seq   MAPSVDSIATSVANSLSNGL.......AGDVRKKTSGAGSLLPTTETTQIDIVERILADAGATDQIKLDGYTLTLGDVVGAARHGRTVKVADSPQIREKIDASVEFLRTQLDNSVYGVTT   113
Consensus           mapsvdsiatsvanslsngl        gdv kkt gagsllpttettq diveriladagatdqikldgytltlgdvvgaar gr vkvadsp irekidasveflrtqldnsvygvtt

AA-KF770992.1.seq   GFGGSADTRTEDAISLQKALLEHQLCGVLPTSMDGFALGRGLENSLPLEVVRGAMTIRVNSLTRGHSAVRIVVLEALTNFLNHGITPIVPLRGTISASGDLSPLSYIAASITGHPDSKVH   240
AA-MG712805.1.seq   GFGGSADTRTEDAISLQKALLEHQLCGVLPTSMDGFALGRGLENSLPLEVVRGAMTIRVNSLTRGHSAVRIVVLEALTNFLNHGITPIVPLRGTISASGDLSPLSYIAASITGHPDSKVH   233
Consensus           gfggsadtrtedaislqkallehqlcgvlptsmdgfalgrglenslplevvrgamtirvnsltrghsavrivvlealtnflnhgitpivplrgtisasgdlsplsyiaasitghpdskvh

AA-KF770992.1.seq   VDGKIMSAQEAIALKGLQPVVLGPKEGLGLVNGTAVSASMATLALTDAHVLSLLAQALTALTVEAMVGHAGSFHPFLHDVTRPHPTQIEVARNIRTLLEGSKYAVHHETEVKVKDDEGIL   360
AA-MG712805.1.seq   VDGKIMSAQEAIALKGLQPVVLGPKEGLGLVNGTAVSASMATLALTDAHVLSLLAQANTALTVEAMVGHAGSFHPFLHDVTRPHPTQIEVARNIRTLLEGSKYAVHHETEVKVKDDEGIL   353
Consensus           vdgkimsaqeaialkglqpvvlgpkeglglvngtavsasmatlaltdahvlsllaqa taltveamvghagsfhpflhdvtrphptqievarnirtllegskyavhhetevkvkddegil

AA-KF770992.1.seq   RQDRYPLRCSPQWLGPLVSDMIHAHAVLSLEAGQSTTDNPLIDLENKMTHHGGAFMASSVGNTMEKTRLAVALMGKVSFTQLTEMLNAGMNRALPSCLAAEDPSLSYHCKGLDIAAAAYT   480
AA-MG712805.1.seq   RQDRYPLRCSPQWLGPLVSDMIHAHSVLSLEAGQSTTDNPLIDLENKMTHHGGAFMASSVGNTMEKTRLAVALMGKVSFTQLTEMLNAGMNRALPSCLAAEDPSLSYHCKGLDIAAAAYT   473
Consensus           rqdryplrcspqwlgplvsdmihah vlsleagqsttdnplidlenkmthhggafmassvgntmektrlavalmgkvsftqltemlnagmnralpsclaaedpslsyhckgldiaaaayt

AA-KF770992.1.seq   SELGHLANPVSTHVQPAEMGNQAINSLALISARRTAEANDVLSLLLATHLYCVLQAVDLRAMEFEHTKAFEPMVTELLKQHFGALATAEVEDKVRKSIYKRLQQNNSYDLEQRWHDTFSV   600
AA-MG712805.1.seq   SELGHLANPVSTHVQPAEMGNQAINSLALISARRTAEANDVLSLLLATHLYCVLQAVDLRAMEFEHTKEFEPMVTALLKQHLGALATADVEDKVRKSIYKRLQQNNSYDLEQRWHDTFSV   593
Consensus           selghlanpvsthvqpaemgnqainslalisarrtaeandvlslllathlycvlqavdlramefehtk fepmvt llkqh galata vedkvrksiykrlqqnnsydleqrwhdtfsv

AA-KF770992.1.seq   ATGAVVEALAGQEVSLASLNAWKVACAEKAIALTRSVRDSFWAAPSSSSPALKYLSPRTRVLYSFVREEVGVKARRGDVYLGKQEVTIGTNVSRIYEAIKSGRIAPVLVKMM          712
AA-MG712805.1.seq   ATGAVVEALAGNEVSLASLNAWKVACAEKAIALTRTVRDSFWAAPSSSSPALKYLSPRTRILYSFIREDVGVKARRGDVYLGKQEVTIGTNVSRIYEAIKDGRIAPVLVKMM          705
Consensus           atgavvealag evslaslnawkvacaekaialtr vrdsfwaapsssspalkylsprtr lysf re vgvkarrgdvylgkqevtigtnvsriyeaik griapvlvkmm
```
